# A Novel Method for the Enhancement of Sunflower Growth from Animal Bones and Chicken Feathers

**DOI:** 10.3390/plants13172534

**Published:** 2024-09-09

**Authors:** Ume Laila, Mishkat ul Huda, Isha Shakoor, Aisha Nazir, Muhammad Shafiq, Firdaus e Bareen, Kamran Shaukat, Talha Mahboob Alam

**Affiliations:** 1 Environmental Biotechnology Laboratory, Institute of Botany, University of the Punjab, Lahore 54000, Pakistan; ume.laila1012@gmail.com (U.L.); mishkaatulhuda@gmail.com (M.u.H.); ishashakoor07@gmail.com (I.S.); aisha.botany@pu.edu.pk (A.N.); mshafiq.botany@pu.edu.pk (M.S.); firdaus.botany@pu.edu (F.e.B.); 2 Institute of Molecular Biology and Biotechnology, University of Lahore, Lahore 54000, Pakistan; 3 Centre for Artificial Intelligence Research and Optimization, Design and Creative Technology Vertical, Torrens University Australia, Ultimo, NSW 2007, Australia; 4 Department of Computer Science, Norwegian University of Science and Technology, 7034 Trondheim, Norway

**Keywords:** bone char, feathers-derived biochar, chicken feathers, biochar composite, sunflower

## Abstract

The present study aimed at converting meat industry waste, particularly waste bones and chicken feathers, into biochar to recycle valuable nutrients present in it, which ultimately become part of the municipal waste. The bone biochar (BB) and feathers biochar (FB) were prepared at 550 °C, and their potential was evaluated as an organic amendment for the growth of sunflower. The ash content (AC) and fixed carbon (FC) improved significantly in prepared biochars as compared to raw feedstock. Fourier transform infrared spectroscopy (FTIR), scanning electron microscopy (SEM) and energy dispersive X-ray spectroscopy (EDX) analyses signaled the occurrence of various functional groups viz. amide group and hydroxyapatite, porosity, and multiple nutrients. Application of BB and FB in potted soil alone as well as in composites (1:1, 1:2, 2:1) at 1%, 3%, and 5% (*w*/*w*) and synthetic fertilizer significantly increased soil pH, electrical conductivity (EC_e_), organic matter (OM) and water holding capacity (WHC), while reducing the bulk density (BD). The growth of plants grown in soil treated with a 2:1 composite of feathers and bone biochar at 5% application rate showed significantly greater differences in plant height, total chlorophyll content, and plant dry weight than the control but was comparable to growth with chemical fertilizer, rendering it a potential alternative to chemical-based synthetic fertilizer.

## 1. Introduction

The population expansion has massively increased food demand globally, especially meat [1]. Globally, a 40 million-metric-ton increase is expected in the production of meat within a decade [2]. The increased consumption of meat ends up producing millions of tons of animal-based wastes such as bones, especially in countries with high populations like Pakistan, where bones enriched with plenty of minerals and nutrients are usually discarded and unfortunately become part of solid waste [3]. Recently, the poultry industry attracted the interest of meat consumers [4]. The consumption of chicken rises daily; as a result, a large amount of poultry feathers is generated as a by-product. This quantity is estimated to be 8.5 billion tons per annum globally [5]. Owing to the recalcitrant nature of feathers, these are considered a major pollutant. Keratin makes up 90% of feathers, making them a potential source of numerous amino acids and nutrients when used as an organic amendment [6].

Converting such waste into biochar through pyrolysis would be an appropriate option to reduce its huge volume and would offer a prospective amendment for the sake of heavy metal stabilization in contaminated soils and for elevating soil fertility because of its high Ca, P, N, K, and Mg contents [7]. Biochar refers to a carbonized porous biomass produced from a wide variety of organic biomasses through thermochemical decomposition under limited provision of oxygen [8,9,10]. Feedstock type and synthesis parameters, mainly temperature of pyrolysis, determine biochar properties. Normally, a high pyrolysis temperature results in porous biochar with macropores (200 nm) which provide a condusive environment for the beneficial microorganisms in soil [11,12]. Biochar as an organic soil amendment that enhances the soil nutrient content and its bioavailability, facilitates microbial activity, improves water holding capacity and aeration, decreases bulk density, maintains the structure of soil aggregates, and decreases leaching by changing the pH and enhancing the CEC of soil [13].

Various studies have explored the feasibility of bone char or bone-derived biochar as an immobilizing agent for various toxic metals, such as Cd, Zn, Pb, Hg and As, in polluted water and soil [14,15] and as a sorbent for various herbicides to prevent their leaching [16]. Bone biochar has been reported to elevate the level of Olsen-P or plant-available phosphorus in phosphorus-deficient soils which makes it a potential organic fertilizer [17,18,19,20]. Um-e-Laila et al. [3] reported improvement of growth and yield of ridge gourd after addition of a bone char and rice biochar composite. In contrast, chemical fertilizer has been extensively employed in agricultural land in order to enhance plant productivity. However, excessive application of chemical fertilizer has hazardous impacts on the environment including low soil fertility, ground water pollution, and soil acidification, ultimately causing low efficacy for macro- and micronutrient uptake in plants [21,22].

In various studies, chicken feather biochar has been used to study the adsorption process. The findings showed that the larger surface area, as well as numerous surface functional groups of the biochar, played a key role in the mechanism of adsorption. Chicken feathers can also be used as fertilizer as they contain approximately 15% nitrogen [1]. Sobucki et al. [23]’s hydrolyzate prepared from poultry feathers was reported to elevate the chlorophyll content and dry biomass of roots and leaves of lettuce compared to urea. The findings indicated that the compost derived from poultry feathers using *Streptomyces* sp. increased the available and total nitrogen content of compost and improved the growth of okra [24]. Feather compost was reported to contain various nutrients including N, Mg, P, C, H, Ca, Mn, and SO_4_^2−^ and improved the growth of *Catharanthus roseus* [25].

Various research work has been undertaken to explore the effect of bone char on plant growth, but a limited amount of work has been reported regarding the efficacy of feather biochar. Furthermore, the composite of bone char and feather biochar as rich sources of phosphorous and nitrogen, respectively, on soil health and crop productivity has not yet been studied. This current study aimed to: (1) captivate the potential feasibility of animal-based biochar, such as bone char and feather biochar, through exploration of their various physical and chemical parameters; and (2) evaluate the individual as well as combined impact of bone char and feather biochar in comparison to chemical-based inorganic fertilizer on soil attributes and sunflower growth.

## 2. Results

### 2.1. Characterization of Biochar

The biochar yield was 61.1% and 34.5% for bone biochar and feather biochar, respectively, prepared at 550 °C pyrolysis temperature. The results of proximate analysis of raw feedstock and prepared biochar are given in Table 1. The MC and VC of prepared bone biochar were 7% and 4%, respectively, and for feather biochar 8% and 19%, respectively, which were significantly lower than the moisture content of their respective raw feedstocks. The AC and FC of bone biochar were 32% and 44%, respectively, and for feather biochar 22% and 49%, respectively, which were higher than the ash content as well as fixed carbon of the raw feedstocks [26].

The results of the physico-chemical analysis (Table 2) indicated that pH and EC_e_ of the prepared biochars were higher than that of their respective feedstocks. The pH value for bone char was recorded as 8.92 and for feather biochar 9.06, while the values of EC_e_ were 460 and 537 μS cm^−1^ for bone and feather biochar, respectively. However, the bulk density of the prepared biochar decreased through pyrolysis, with 0.46 g cm^−3^ and 0.26 g cm^−3^ for bone and feather biochar, respectively. The CEC value of feather biochar (8.68 cmol_c_ kg^−1^) was higher than bone char (7.07 cmol_c_ kg^−1^). The results of the nutrient analysis revealed that both biochars have sufficient amounts of nutrients, mainly N, Ca, K, Mg, and P. Bone char had higher levels of P (0.55 g kg^−1^) and Ca (0.68 g kg^−1^), while feather biochar exhibited higher quantities of N (0.63 g kg^−1^), K (0.065 g kg^−1^), and Mg (0.048 g kg^−1^), also supported by EDX analysis.

**Figure 1:**
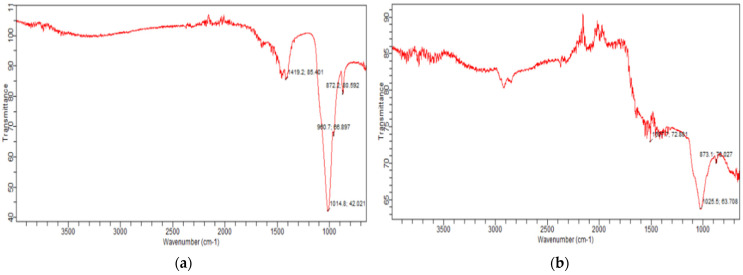
The FTIR spectra of (**a**) bone char and (**b**) feather biochar.

The FTIR analysis indicated the presence of important functional groups in both biochars within the range of 650 cm^−1^ to 4000 cm^−1^. For bone char (Figure 1a), peaks were observed at 872.2 cm^−1^, 960.7 cm^−1^, 1014.8 cm^−1^ and 1419.2 cm^−1^. The wavenumbers 872.2 cm^−1^ and 1419.2 cm^−1^ represented the substitution of CO_3_^2−^ and asymmetric stretching of CO_3_^2−^, respectively [27]. However, peaks at 960.7 cm^−1^ and 1014.8 cm^−1^ indicated the occurrence of a PO_4_^−3^ bond, which represented hydroxyapatite [2]. For feather biochar (Figure 1b), peaks were detected at 873.1 cm^−1^, 1025.5 cm^−1^, and 1507.7 cm^−1^ which indicated the presence of the aromatic –CH group [14], a medium band of -C-OH representing alcoholic or phenolics groups, [13] and Amide II linkage, respectively.

**Figure 2:**
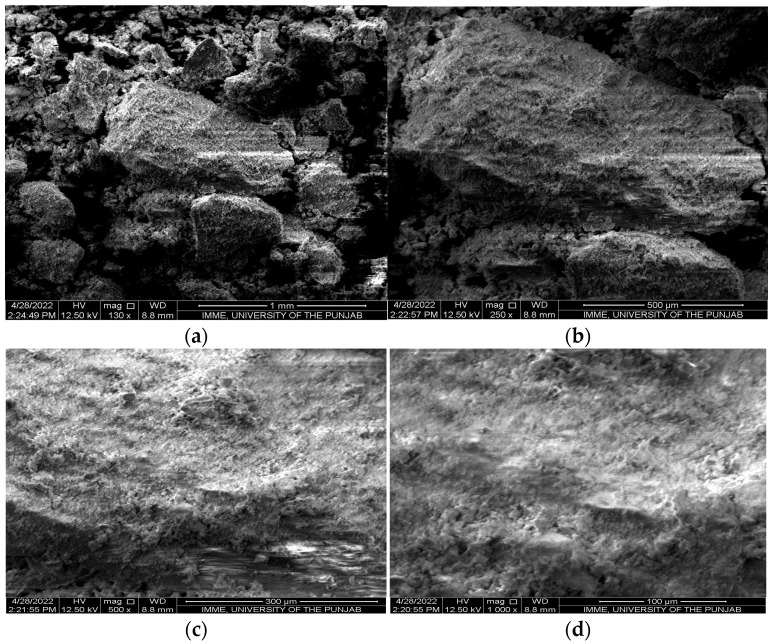
Scanning electron micrographs of bone biochar (BB) at (**a**) 130×, (**b**) 250×, (**c**) 500× and (**d**) 1000×.

**Figure 3:**
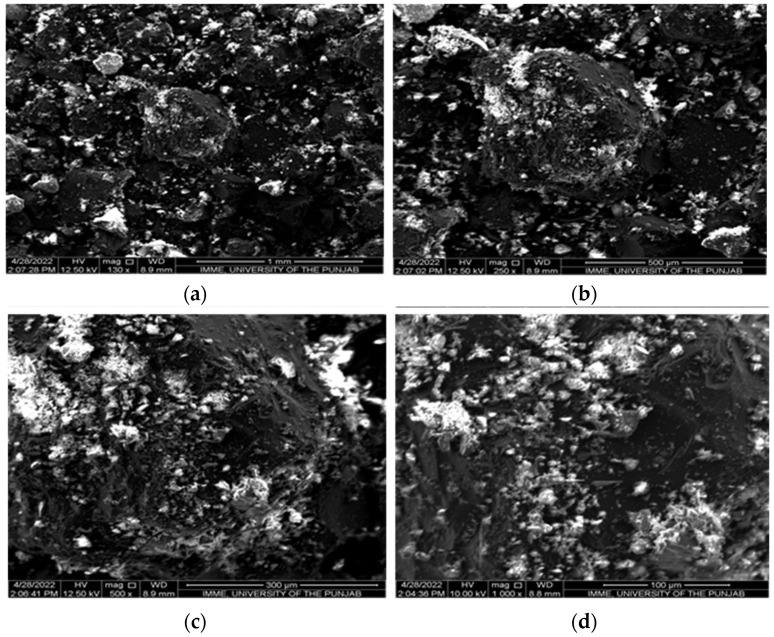
Scanning electron micrographs of feather biochar (FB) at (**a**) 130×, (**b**) 250×, (**c**) 500× and (**d**) 1000×. Arrows are indicating the pores.

Scanning electron micrographs of bone biochar (Figure 2) and feather biochar (Figure 3) were captured at different magnifications, i.e., 130×, 250×, 500×, and 1000×. At lower magnifications, i.e., 130× and 250×, the granular structure of bone char was observed while at higher magnifications, i.e., 500× and 1000×, bone char appeared to have a cottony surface. The surface of feather biochar also exhibited granular structures at lower magnifications, while at magnification 500×, pores were observed [28].

**Figure 4:**
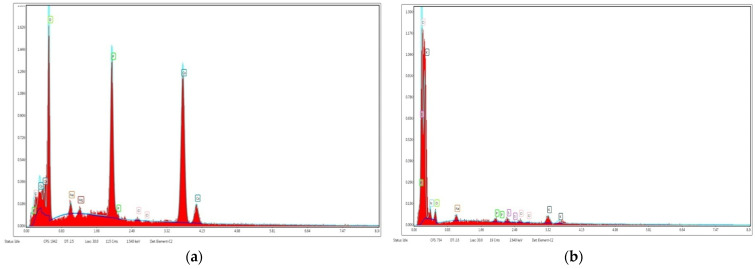
EDX spectra of bone biochar (**a**) and feather biochar (**b**).

EDX analysis of BB and FB was performed to confirm the presence of major elements (Figure 4). The main constituents of bone biochar indicated by EDX spectra (Figure 4a) included P, Ca, and O with atomic weights 13.45%, 25.83% and 58.93%, respectively, mass 17.06%, 42.46%, and 38.66%, respectively, and trace quantities of Na, Mg, and Cl. Feather biochar was revealed (Figure 4b) to have N, K, and O mainly with atomic weights 40.5%, 10.88%, and 33.5%, respectively, while P, Na, S, and Cl were also found in small quantities.

### 2.2. Biochar Effect on Soil Characteristics

The soil amended with bone and feather biochar appeared to have altered properties. The pH, EC_e_, cation exchange capacity (CEC), OM, WHC, and BD were determined for soil supplemented with biochar (Table 3). After 8 days of incubation, pH, EC_e_, CEC, OM, and WHC of soil increased significantly. The application of biochar composites was found more effective at medium (3%) and high (5%) application rates than biochar alone. At a higher application rate (5%), pH values of composites M_1_, M_2_, and M_3_ were 8.94, 9.25 and 8.66, respectively; values of EC_e_ were 143.4 μS cm^−1^, 154.2 μS cm^−1^ and 157.3 μS cm^−1^, respectively, while values of CEC were 21.6, 22.9 and 24.6 cmol_c_ kg^−1^, respectively. However, application of both biochars separately exhibited higher values for pH and EC_e_ in comparison to the control and synthetic fertilizer [29]. The values of CEC in all treatments increased with increasing quantity of biochar; likewise, a significant increase was recorded in OM and WHC values with increasing quantity of biochar, while a significant reduction was noted in soil bulk density after the incorporation of biochar because of the lower weight of prepared biochar.

After harvesting the 78-day-old sunflowers, the values of pH, EC_e_, CEC, OM, and WHC obtained from soils treated with composites showed a significant improvement compared to other biochar treatments, control, and chemical fertilizer-supplemented soil (Table 4). Feather biochar and all combinations of biochar composite appeared to improve values of pH, EC_e_, CEC, OM, and WHC, while the values of BD decreased further. At the higher application rate (5%), pH values of composites M_1_, M_2_, M_3_ increased to 9.32, 9.35, and 8.70, respectively, values of EC_e_ were 217.4 μS cm^−1^, 226.6 μS cm^−1^ and 262.6 μS cm^−1^, and CEC increased to 22.4, 23.6 and 25.6 cmol_c_ kg^−1^, respectively.

### 2.3. Biochar Effect on Sunflower Growth

**Figure 5:**
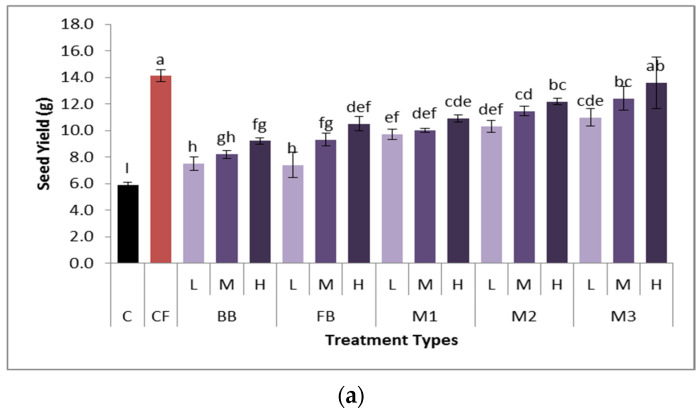

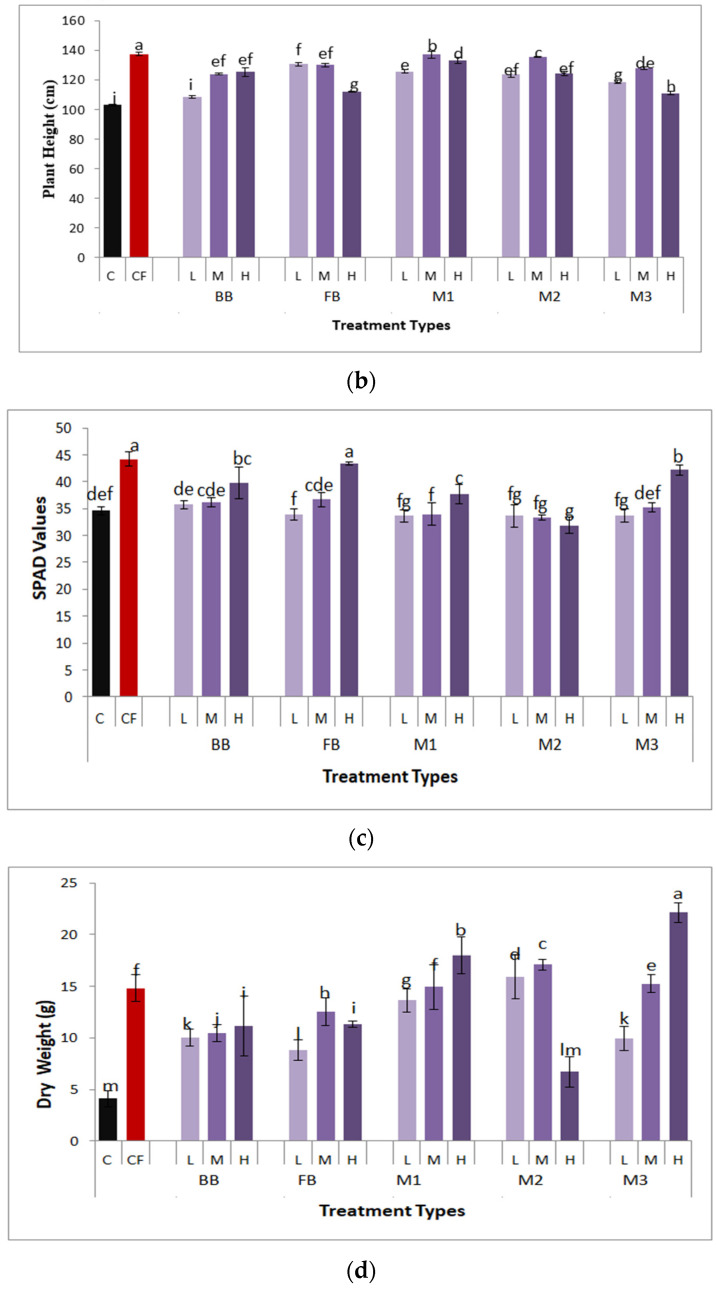
Variations in growth measurements. (**a**) Seed yield, (**b**) plant height, (**c**) SPAD values, and (**d**) plant dry biomass of sunflowers cultivated under different application levels of bone char (BB), feather-derived biochar (FB) and biochar composites (M1, M2, M3) along with control and commercial fertilizer. The values represented with similar letters are statistically not different, with reference to Duncan’s multiple range test (*p* = 0.05). The bar represents the standard error.

The growth parameters of 78-day-old sunflowers were recorded. Both biochar alone and biochar composite-treated soils exhibited the maximum plant dry biomass and seed yield compared to the control. All biochar treatments showed significantly higher crop yield compared to the control. The treatment M3, i.e., composite_(1:2)_ specifically, exhibited maximum seed yield at all doses (Figure 5a). However, the application of composite biochar at a high dosage (M3H) showed pronounced seed yield (13.6 g) which was not significantly different from the commercial fertilizer treatment (14.1 g). The biochar composites at 3% application rate exhibited the maximum plant height. The trend for plant height (Figure 5b) can be given as CF > M_1_M > M_2_M > M_1_H > M_2_M.

Treatments FBH, M_3_H and BBH exhibited significantly higher values for total SPAD values than the control but equivalent to chemical fertilizer-treated soil. The SPAD values were significantly higher in the case of FB-H (42.3) and CF (42) (Figure 5c). The treatments M3.H and M1.H (22.5 g and 18.5 g, respectively) had significantly higher dry biomass than that of the CF treatment (Figure 5d). The trend for maximum sunflower plant dry biomass (Figure 5d) was M_3_H > M_1_H > M_2_M > M_2_L > M_3_M > CF. In contrast, the =-control showed the lowest values for all the parameters. At the time of the harvest, i.e., after 61 days of the experiment, plants from only a few treatments developed flowers. The early flowering was observed for the sub-treatments BBH, FBL, FBM, FBH, M_1_L, M_1_M, M_1_H and M_3_H, along with the commercial fertilizer. The plants grown in soil treated with feathes biochar and composite 1 exhibited early flowering at all application rates, i.e., (1%, 3% and 5%), while bone char and composite 3 induced early flowering at higher application rates, i.e., (5%). The control and composite 2 (M2) showed late inflorescence. Overall, the higher application rate was found effective in producing flowers early. The flowering results observed in the higher application rates (5%) of BBH, FBH and M3H were significantly greater than in the control and commercial fertilizer treatment.

## 3. Discussion

The biochar yield (Table 1) was recorded as 61% and 34.51% for bone biochar and feather biochar, respectively. The yield is mainly dependent on the pyrolysis temperature. It has been reported that a decrease in biochar yield was observed by increasing the temperature of pyrolysis, mainly due to the loss of moisture, thermal degradation of hydrocarbons and loss of volatile compounds during the process of pyrolysis [12]. This also explains the reason behind lower moisture and volatile contents of prepared biochar than raw feedstock [30,31,32]. The increased percentage of ash content (Table 1) at a high temperature is because of the removal of volatiles, and accumulation of organic residues and mineral content [10] and the increase in fixed carbon percentage (Table 1) might be due to the accumulation of recalcitrant carbon and mineral contents resulting from high temperature of pyrolysis.

Physicochemical properties (Table 2), such as pH and EC, are useful indicators of biochar quality. The increase in pH and EC_e_ values is dependent upon the type of raw biomass and the temperature of pyrolysis. The increase in pH of prepared biochar might indicate the presence of a basic group. It was reported that the rise in the pH value upon increasing temperature is because of separation of alkali ions from various organic compounds and the accumulation of basic functional groups [33]. The increased EC_e_ values indicated the existence of soluble salts in higher quantities [34]. The CEC of biochar is an indication of its capacity to adsorb cations. The feather biochar has higher CEC than bone biochar. It might be because of the functional groups present on the surface of biochar such as phenolics. These groups impart cation exchange capacity to biochar [35]. The nutrient analysis showed that both bone and feather biochars contain important macro- and micronutrients. These findings are supported by other studies [3,36].

The peak at wavenumber 1419.2 cm^−1^ of the FTIR spectrum of bone char (Figure 1a) indicated asymmetric stretching of CO_3_^2−^ representing carbonates entering into the lattice of the biochar. The representation of the peak at 872 cm^−1^ was also an indication of the substitution of the CO_3_^2−^ ion. These findings are supported by Akindoyo et al. [37] and Piccirillo et al. [38]. The peak appearing at 1014.8 cm^−1^ indicated the asymmetric stretching of PO_4_^−3^, and the peak at 960.7 cm^−1^ also indicated PO_4_^−3^ representing hydroxyapatite presence [2,39]. In the feather biochar FTIR spectrum, the peak appearing at 1025.5 cm^−1^ hinted a medium band of -C-OH representing the occurrence of an alcoholic or phenolic group, contributing to the moisture content of biochar [11]. The exhibition of a peak at 873.1 cm^−1^ indicated the occurrence of the aromatic –CH group, in agreement with the study of Huang et al. [14], and the peak appearing at 1509.7 cm^−1^ proved the occurence of Amide II linkage [40].

The SEM microphotographs revealed the surface morphology of both bone biochar (Figure 2) and feather biochar (Figure 3). The bone biochar at the lower magnification of 130× appeared in bulky granular form while higher magnification revealed a rough surface of biochar particles with a cottony appearance having no pores. The findings of Alkurdi et al. [2], where a flat pore-less surface was observed for bone char produced at 500 °C, are in agreement with the current findings. The biochar prepared from feathers also appeared granular at 130×, while at 500× pores of varying size were observed. The pores in biochar enable it to hold greater amounts of nutrients [41]; hence, it can be deduced that the feather biochar has the ability to hold more nutrients than bone biochar.

The EDX analysis of bone char (Figure 4a) revealed the high percentages of constitutive components of hydroxyapatite, i.e., Ca, O and P. The results are supported by the study of Akindoyo et al. [37]. Other elements included Na, Mg, Al, Si, and Cl. The elemental composition of feather biochar (Figure 4b) included N, K, P, Ca, O, and trace amounts of Na, Mg, Zn, S, Al, Mo, and Cl. The EDX analysis provided a confirmation of existence of important minerals in both biochars which upon transferring to soil will elevate the attributes of soil and positively affect plant growth.

The soil pH, EC_e_, CEC, OM, and WHC (Table 3) increased with the increased application rates of biochar and are in agreement with the findings of Furtado et al. [42] and Sikder and Joardar [43]. The rise in soil pH could be due to the dissolution of carbonates, hydroxides and oxides originating from the basic components of biochar. The increase in soil EC_e_ is the result of dissolution of salts present in the biochar, while the increase in soil CEC upon adding biochar is because of the release of free basic cations, such as Mg^+2^, K^+^, and Ca^+2^, into the soil aqueous phase [44]. Both bone and feather biochars had bulk density lower than that of the raw material, indicating that biochar is a light-in-weight substance and had significantly lower density than soil. The reason might be that the addition of biochar lowered the bulk density of soil by combining with soil particles to form soil aggregates. Further, biochar addition created more pore spaces, reducing the bulk density and providing more space to hold nutrients [45]. Similar results were recorded by Burrell et al. [46]. The increase in porosity and pore continuity increased the WHC of soil. Micropores and mesopores contain the plant available water [36]. These results are are in accordance with the study of Verheijen et al. [47].

The relatively higher CEC value of feather biochar suggests that the soil’s ability to adsorb nutrients under feather biochar treatments will increase, decreasing nutrient leaching; hence, more nutrients will be available to the plant [36]. Biochar is a rich source of organic matter and upon addition to soil, it increased the soilorganic matter. Soil organic matter provides an indication of soil fertility [48]. Soil organic matter (SOM) acts as a primary binder for water stable aggregates of soil. These findings are consistent with the study of Cen et al. [49] that argues that SOM increases with the increased application rate of biochar. The improvement in the physical characteristics of soil may lead to enhanced microbial activity in soil, leading to improved defense mechanisms in plants.

After the harvest of 61-day-old sunflowers, the values obtained (Table 4) from the biochar composites improved significantly compared to the control and commercial fertilizer-treated soil. The biochar composite (2:1) significantly improved soil properties, indicating that the combination of both biochars transferred valuable macro- and micronutrients to the soil. A previous study also reported the gradual improvement of soil properties over time after biochar application [3]. Bone char is a rich source of Ca and P and has been proven to be a source of P to beneficial fungi when grown solely on bone char particles [50]. Chicken feather biochar might have enhanced the available content of soil N and P. The improved soil attributes, such as increased soil pH, WHC and availability of nutrients, could enhance soil microbial activity [51], and it is assumed that this increased activity has led to increased availability of macro- and micronutrients to plants through the process of soil microbial solubilization [52].

The impact of biochar treatments on sunflower growth was evaluated comparing different growth parameters statistically. In general, soil supplemented with biochar accelerated the seed yield (Figure 5a) compared to the control and commercial fertilizer-treated soil. The treatment M3 had maximum seed yield in all the treatments. The improvement in soil WHC and porosity by composite biochar application led to improved soil moisture and soil aeration and nutrient availibility; hence, elevating the seed yield. Moreover, some biochars produce ethylene even in the absence of soil microbial inoculation that plays a major role in early inflorescence and ultimately high crop yield [53].

The biochar treatment increased plant height (Figure 5b) significantly compared to the control but did not exceed the length of plants treated with commercial fertilizer. By increasing the biochar input from 1% to 3%, plant height showed an increasing trend compared to control, consistent with the study of Chen et al. [54], but at the higher dose (5%), the plant height reduced. A possible reason for this could be biomass allocation; the implementation of biochar enhanced the leaf biomass and lessened the stem biomass allocation [55]. A previous study reported a positive correlation between the stem biomass and plant height [56,57].

The SPAD values (Figure 5c) are indicators of plant health; a high chlorophyll content means a healthy plant [58]. The findings of this study indicted a significant effect of BBH, FBH and M_3_H-treated soil on total chlorophyll content, and it might be because of the elevated levels of plant available nutrients, mainly N, P, and Mg, due to the slow-release nature of biochar. N and Mg are the factors responsible in regulating the photosynthetic activities of plants as these elements are the constituent elements of chlorophyll [59]. A significant difference was recorded in the dry weight of sunflower (Figure 5d) amended with biochar composite compared to the control and fertilizer [60]. The early flowering in some treatments might be the result of availability of more nutrients to the plant. That could happened because of the increased nitrogen availability as indicated by the FTIR and EDX analysis of feather biochar. As nitrogen is the constitutive element of chlorophyll and plant enzymes, an increased nitrogen concentration improves crop yield [58].

The findings of this study showed that the biochar addition both alone and in composites significantly improved plant growth compared to the control and was nearly equivalent to the commercial fertilizer. In the biochar alone application, treatment BT_2_ (feather biochar) showed the best results while in composites, treatments BT_3_ and BT_4_ were found to be effective in improving plant growth. Their values were significantly higher than the control and comparable to the commercial fertilizer, indicating that biochar might have imparted diverse and a number of surfaces to soil with a vast range of electrostatic charges which possibly transformed it into a good source of nutrients. This could be one of the reasons for improved plant growth comparable to the commercial fertilizer.

The findings of this study showed that bone and feather biochars and their composites in different ratios had a significant impact on plant growth, comparable to commercial fertilizer, by changing soil characteristics and improving the amount of bio-available nutrients in soil. The results of advanced analyses are evident of the presence of various valuable functional groups and minerals in both biochars which contributed to improving the soil properties and plant growth.

## 4. Materials and Methods

### 4.1. Collection and Preparation of Experimental Materials

Here, two animal-based feedstocks, waste bones (mutton and beef bones) and chicken feathers were used to prepare two different biochars. The bones were collected from a famous food street of Lahore (31°34′00.7″ N and 74°18′34.0″ E) while the chicken feathers were procured from the poultry market of Lahore (31°32′34.5″ N and 74°19′11.8″ E). The feedstocks were then washed and air-dried. The waste bones were cut into uniform-sized pieces of 2 cm using an electric cutter. Both feedstocks were then pyrolyzed, separately, at 550 °C for 25 min residence time under a highly restricted supply of oxygen by obtaining 0.001 Torr pressure in the feedstock chamber of the semi-automated pyrolyser with a ramp rate of 30 °C per min. Each biochar was then crushed and passed through a mesh size of 0.5 mm before using in the experiment. The calculations for biochar yield were performed according to the given formula:

Yield (%) = W_RF_/W_BC_ × 100

where

W_RF_ = Mass of raw feedstock
W_BC_ = Mass of prepared biochar

Soil (having pH 8.06, organic matter 3.14%, 0.11 g N kg^−1^,0.036 g P kg^−1^, 0.331 g K kg^−1^, and bulk density 1.35 g cm^−3^) used in the study was collected from the Botanical Garden of University of the Punjab with no history of metal toxicity. Sunflower was used as a test plant in this study. The certified sunflower seeds of AGUARA 4 variety were purchased from Sky Seeds Store.

### 4.2. Characterization of Biochar

Proximate analysis, including moisture content (MC), ash content (AC), and volatile content (VC) of raw feedstock and derived biochar, were performed following the standard procedure [26]. The fixed carbon was recorded through the following formula:

FC (%) = 100 − (MC (%) + VC (%) + AC (%))

The pH, electrical conductivity, total dissolved solids and bulk density were determined by following the procedure described in a previous study [3]. The extract was prepared in 1:20. CEC of biochar was determined by employing ammonium acetate solution as per the protocol provided by Chapman [27]. For nutrient estimation, samples were digested by preparing aqua regia of HClO_4_:HNO_3_ (4:1). The acid digestion was performed in a glass fume hood, and nutrients in digested biochar samples were assessed through flame spectrophotometer (Model: PFP7 and PFP7/C JENWAY) [28].

The morphology of the surface of each biochar was studied through scanning electron microscopy (SEM) (Model No. SEM. JSM 6480LV, Freising, Germany). The mineral composition of the surface of the biochars was studied through energy dispersive X-ray analysis. The detection of functional groups present in biochar was performed through Fourier transformation infrared spectroscopy (FTIR) (IR prestige-21 shimadzu, Kyoto, Japan).

### 4.3. Experimental Set-Up

A pot experiment was planned by employing a randomized complete block design (RCBD) in the Botanical Garden of University of the Punjab, Lahore, with each treatment having three replications. Pots of 2.5 kg capacity were used in the experiment. The treatments are as follows:

- Control (soil with no amendment or fertilizer).
- Soil treated with chicken feather biochar (*w*/*w*) at three application rates (1%, 3%, and 5%).
- Soil treated with bone char (*w*/*w*) at three application rates (1%, 3%, and 5%).
- Soil amended with composite (M_1_) of chicken feather biochar and bone char with 1:1 (*FB + BB: w*/*w*) at three application rates (1%, 3%, and 5%).
- Soil amended with composite (M_2_) of chicken feather biochar and bone char with 1:2 (*FB + BB: w*/*w*) at three application rates (1%, 3%, and 5%).
- Soil amended with composite (M_3_) of chicken feather biochar and bone char with 2:1 (*FB + BB: w*/*w*) at three application rates (1%, 3%, and 5%).
- Inorganic commercial fertilizer (NPK dosage corresponding 150:70:50 kg h^−1^).

Before sowing, each potted soil was mixed with biochar, moistened, and left for 8 days for a pre-incubation period to enhance the chemical reactions between soil and biochar. After 8 days, 5 sunflower seeds were sown in each pot and all pots were watered regularly. Rate of germination was assessed after 10 days of sowing. One healthy and vigorous seedling was retained in each pot. The growth measurements were recorded after the growing period of 78 days.

### 4.4. Soil Analysis

The soil samples from each pot were collected from cores before sowing and after harvesting. The collected samples were air-dried, crushed and sieved through a sieve of pore size < 2 mm. Soil pH, EC_e_, CEC and bulk density were determined by following the procedure mentioned above. The soil organic matter was determined by the loss-on-ignition method. Soil water holding capacity was determined by following the procedure of Gessert [29].

### 4.5. Growth and Yield Measurements

The impact of different amendments on plant growth parameters including plant height, dry weight and SPAD values of plants were detected at the time of harvest of 61-day-old sunflowers.

### 4.6. Statistical Analysis

One way ANOVA (Microsoft Excel 9.3 version) was employed for statistical analyses of the obtained data. Means and standard deviation were calculated, and comparison among different treatment means was performed using least significant differences (*p =* 0.05).

## 5. Conclusions

The recycling of animal-based wastes, such as bones and feathers, through pyrolysis provides a sustainable solution for waste management issues and gives a valuable product of agricultural importance, i.e., biochar. The implementation of such biochar and their composites (as sources of macronutrients) in soil resulted in early flowering and higher seed yield comparable to commercial fertilizer, thus providing a potential alternative to commercial fertilizer. The feather biochar showed better results than the bone char and composite 1:1. However, the composite 2:1 gave better outcomes than other biochar treatments. Overall, the application of feather biochar and bone biochar composite_2:1_ (M_3_) at higher application rates (5%) incremented sunflower growth and soil physico-chemical properties as an organic soil ammendment.

Further research work can be carried out on the influence of feather and bone biochar on the microbial dynamics of soil; a comparative study of feather biochar with a plant-based biochar and their composite can also be designed. Moreover, the long-term influence of biochar composites on various soils can be evaluated.

## Figures and Tables

**Table 1:** Proximate analysis of raw bones (RB), raw feathers (RF), bone biochar (BB) and feather biochar (FB).

		RB	RF	BB	FB
**Biochar Yield**				61.1 ± 0.28	34.5 ± 0.01
**Proximate Analysis (wt. %)**	**MC%**	27 ± 0.32	20 ± 0.75	7 ± 0.01	8 ± 0.01
	**VC%**	24 ± 0.01	41 ± 0.5	4 ± 0.01	19 ± 0.34
	**AC%**	15 ± 0.60	10 ± 0.1	32 ± 0.57	22 ± 0.51
	**FC%**	33 ± 1.12	31 ± 2.09	44 ± 1.44	49 ± 0.49

Where; MC: moisture content, VC: volatile content, FC: fixed carbon content, AC: ash content. The mean values were significant at 0.05 level of probability.

**Table 2:** Physico-chemical analysis of bone char (BB) and feather biochar (FB) produced at 550 °C.

		Physico-Chemical Analysis			Nutrients (g kg^−1^)				
Samples	pH	EC_e_ (μS/cm)	BD (g/cm^3^)	CEC (cmol kg^−1^)	N	P	K	Ca	Mg
**BB**	8.92 ± 0.06	460 ± 4.04	0.46 ± 0.02	7.07 ± 0.02	0.39 ± 0.002	0.55 ± 0.002	0.013 ± 0.003	0.68 ± 0.004	0.016 ± 0.003
**FB**	9.06 ± 0.01	537 ± 3.21	0.26 ± 0.002	8.68 ± 0.02	0.63 ± 0.001	0.024 ± 0.002	0.065 ± 0.01	0.054 ± 0.004	0.048 ± 0.002

Where; ECe: electrical conductivity of soil extract, CEC: cation exchange capacity, OM: organic matter, BD: bulk density, N: nitrogen, P: phosphorous, K: potassium, Mg: magnesium, Ca: calcium. The mean values were significant at 0.05 level.

**Table 3:** Physico-chemical properties of soil having three different application rates (L = 1%, M = 3%, H = 5%) of feather biochar (FB) and bone char (BB) alone and in three combinations (FB + BB; M1 = 1:1, M2 = 1:2, M3 = 2:1) after an 8-day incubation period along with control (only soil) and commercial fertilizer (CF).

Treatments	Sub-Treatments	pH	EC_e_ (μS cm^−1^)	CEC (cmol_c_ kg^−1^)	OM (%)	WHC (%)	BD (g cm^−3^)
Control	**C**	8.05 ^hi^ ± 0.01	61.33 ^jk^ ± 0.15	6.6 ^l^ ± 0.65	3.14 ^k^ ± 0.20	44.53 ^j^ ± 1.36	1.39 ^a^ ± 0.01
BT_1_	**BBL**	8.77 ^e^ ± 0.42	64.73 ^j^ ± 1.18	12.5 ^j^ ± 0.90	3.72 ^i^ ± 0.98	48.10 ^h^ ± 0.45	1.36 ^abc^ ± 0.05
	**BBM**	8.61 ^fg^ ± 0.06	59.89 ^l^ ± 0.81	14.4 ^i^ ± 0.73	3.83 ^hi^ ± 0.02	51.93 ^ef^ ± 1.00	1.35 ^abcd^ ± 0.02
	**BBH**	9.14 ^b^ ± 0.04	134.2 ^c^ ± 1.08	17.2 ^h^ ± 0.70	4.16 ^fg^ ± 0.03	61.40 ^d^ ± 0.40	1.32 ^cde^ ± 0.01
BT_2_	**FBL**	8.67 ^f^ ± 0.05	74.06i ± 2.75	16.4 ^h^ ± 0.60	3.17 ^k^ ± 0.50	46.33 ^i^ ± 0.35	1.36 ^abcd^ ± 0.05
	**FBM**	8.53 ^g^ ± 0.04	113.40 ^g^ ± 1.44	19 ^g^ ± 0.85	3.34 ^j^ ± 0.06	53.53 ^g^ ± 0.55	1.30 ^de^ ± 0.01
	**FBH**	9.13 ^b^ ± 0.02	135.40 ^c^ ± 1.54	21.4 ^cde^ ± 0.62	3.63 ^i^ ± 0.06	57.90 ^f^ ± 0.90	1.28 ^e^ ± 0.01
BT_3_	**M_1_L**	8.93 ^cd^ ± 0.05	118.20 ^f^ ± 1.31	18.8 ^g^ ± 0.65	3.99 ^gh^ ± 0.07	59.67 ^e^ ± 0.78	1.37 ^ab^ ± 0.01
	**M_1_M**	8.92 ^cd^ ± 0.04	127.90 ^e^ ± 1.73	20.3 ^ef^ ± 0.40	4.35 ^ef^ ± 0.13	63.13 ^c^ ± 1.00	1.34 ^cde^ ± 0.01
	**M_1_H**	8.94 ^cd^ ± 0.04	143.40 ^b^ ± 1.41	21.6 ^cd^ ± 0.60	4.42 ^de^ ± 0.03	65.76 ^b^ ± 1.00	1.32 ^cde^ ± 0.01
BT_4_	**M_2_L**	8.85 ^de^ ± 0.04	128.60 ^de^ ± 0.47	20.5 ^def^ ± 0.83	4.63 ^bc^ ± 0.16	64.75 ^b^ ± 0.76	1.35 ^abcd^ ± 0.02
	**M_2_M**	8.97 ^c^ ± 0.04	132.20 ^cd^ ± 5.11	21.7 ^c^ ± 0.80	4.79 ^bc^ ± 0.12	65.48 ^b^ ± 0.12	1.36 ^abc^ ± 0.05
	**M_2_H**	9.25 ^a^ ± 0.03	154.20 ^a^ ± 4.76	22.9 ^b^ ± 0.30	4.84 ^a^ ± 0.13	67.09 ^a^ ± 0.07	1.33 ^bcd^ ± 0.02
BT_5_	**M_3_L**	7.98 ^i^ ± 0.12	109.60 ^h^ ± 1.51	20.1 ^f^ ± 0.37	4.33 ^ef^ ± 0.05	64.45 ^b^ ± 0.77	1.37 ^ab^ ± 0.02
	**M_3_M**	8.15 ^h^ ± 0.05	131.70 ^cde^ ± 2.41	23.4 ^b^ ± 0.60	4.59 ^cd^ ± 0.22	65.36 ^b^ ± 0.60	1.36 ^abc^ ± 0.05
	**M_3_H**	8.66 ^f^ ± 0.11	157.30 ^a^ ± 3.07	24.6 ^a^ ± 0.86	4.80 ^ab^ ± 0.05	68.33 ^a^ ± 0.10	1.35 ^abcd^ ± 0.05
CF	**CF**	8.10 ^h^ ± 0.01	143.80 ^b^ ± 0.70	11.7 ^k^ ± 0.28	3.67 ^i^ ± 0.18	47.30 ^g^ ± 1.47	1.38 ^ab^ ± 0.01

ECe: electrical conductivity of soil extract, CEC: cation exchange capacity, OM: organic matter, WHC: water-holding capacity, BD: bulk density. Values are means of three replicates ± SD. Mean values with different letters are significantly different from each other.

**Table 4:** Post-harvest physico-chemical properties of soil having three different application rates (L = 1%, M = 3%, H = 5%) of feather biochar (FB) and bone char (BB) alone and in three combinations (FB + BB; M1 = 1:1, M2 = 1:2, M3 = 2:1) along with the control (only soil) and commercial fertilizer (CF).

Treatments	Sub-Treatments	pH	EC_e_ (μS cm^−1^)	CEC (cmol_c_ kg^−1^)	OM (%)	WHC (%)	BD (g cm^−3^)
Control	**C**	7.90 ^k^ ± 0.11	66.33 ^k^ ± 2.79	6.1 ^l^ ± 0.77	3.53 ^e^ ± 0.01	45.06 ^i^ ± 1.53	1.38 ^a^ ± 0.01
BT_1_	**BBL**	8.43 ^ghi^ ± 0.49	138.20 ^i^ ± 0.77	13.1 ^j^ ± 0.80	3.91 ^cd^ ± 0.08	56.34 ^g^ ± 0.57	1.30 ^cde^ ± 0.03
	**BBM**	8.38 ^ghi^ ± 0.37	133.00 ^j^ ± 0.39	15.3 ^i^ ± 0.40	4.11 ^c^ ± 0.02	67.70 ^e^ ± 1.06	1.29 ^cdef^ ± 0.05
	**BBH**	9.14 ^abc^ ± 0.55	207.40 ^de^ ± 1.49	18.4 ^g^ ± 0.56	4.41 ^b^ ± 0.02	69.78 ^d^ ± 0.93	1.25 ^fg^ ± 0.01
BT_2_	**FBL**	8.68 ^ef^ ± 0.11	149.00 ^h^ ± 2.67	17.1 ^h^ ± 0.30	3.34 ^e^ ± 0.03	56.14 ^g^ ± 0.38	1.31 ^cde^ ± 0.04
	**FBM**	8.56 ^fgh^ ± 0.07	186.90 ^g^ ± 2.56	19.5 ^g^ ± 0.60	3.60 ^de^ ± 0.06	63.48 ^f^ ± 1.27	1.24 ^gh^ ± 0.02
	**FBH**	9.13 ^abc^ ± 0.12	209.50 ^d^ ± 0.71	22.2 ^de^ ± 0.35	3.89 ^cd^ ± 0.07	66.77 ^e^ ± 0.90	1.22 ^h^ ± 0.02
BT_3_	**M_1_L**	9.01 ^bcd^ ± 0.05	191.30 ^g^ ± 1.81	19.4 ^g^ ± 0.72	4.43 ^b^ ± 0.13	68.38 ^de^ ± 0.60	1.34 ^abc^ ± 0.03
	**M_1_M**	8.99 ^cde^ ± 0.20	200.70 ^f^ ± 1.17	21.2 ^ef^ ± 0.65	4.65 ^b^ ± 0.14	72.41 ^cn^ ± 1.42	1.29 ^def^ ± 0.01
	**M_1_H**	9.32 ^ab^ ± 0.06	217.40 ^c^ ± 1.37	22.4 ^cd^ ± 0.80	4.68 ^b^ ± 0.04	76.24 ^b^ ± 0.54	1.27 ^efg^ ± 0.01
BT_4_	**M_2_L**	8.87 ^cdef^ ± 0.03	204.00 ^ef^ ± 0.93	21.3 ^ef^ ± 0.79	4.80 ^ab^ ± 0.08	73.53 ^c^ ± 0.40	1.31 ^bcde^ ± 0.01
	**M_2_M**	8.99 ^cde^ ± 0.10	208.30 ^de^ ± 1.72	23 ^cd^ ± 0.72	4.91 ^ab^ ± 0.03	75.35 ^b^ ± 0.76	1.30 ^cde^ ± 0.02
	**M_2_H**	9.35 ^a^ ± 0.05	226.60 ^b^ ± 5.47	23.6 ^bc^ ± 0.70	5.14 ^a^ ± 0.01	77.01 ^b^ ± 0.70	1.29 ^def^ ± 0.01
BT_5_	**M_3_L**	8.06 ^jk^ ± 0.09	186.30 ^g^ ± 2.64	21 ^f^ ± 0.65	4.55 ^b^ ± 0.06	73.22 ^c^ ± 0.68	1.36 ^a^ ± 0.02
	**M_3_M**	8.27 ^hij^ ± 0.30	224.60 ^b^ ± 4.88	24.3 ^b^ ± 0.87	4.67 ^b^ ± 0.05	76.85 ^b^ ± 0.72	1.35 ^ab^ ± 0.04
	**M_3_H**	8.70 ^defg^ ± 0.03	262.60 ^a^ ± 6.32	25.6 ^a^ ± 0.83	5.28 ^a^ ± 0.06	83.18 ^a^ ± 2.81	1.33 ^abcd^ ± 0.03
CF	**CF**	8.12 ^ijk^ ± 0.04	146.6h ± 0.65	10.8 ^k^ ± 0.35	3.59 ^de^ ± 0.16	54.10 ^h^ ± 0.83	1.37 ^a^ ± 0.01

ECe: electrical conductivity of soil extract, CEC: cation exchange capacity, OM: organic matter, WHC: water-holding capacity, BD: bulk density. Values are means of three replicates ± SD. Mean values with different letters are significantly different from each other.

## Data Availability

Dataset available on request from the authors.
